# Novel botanical drug DA-9803 prevents deficits in Alzheimer’s mouse models

**DOI:** 10.1186/s13195-018-0338-2

**Published:** 2018-01-29

**Authors:** Guillaume J. Pagnier, Ksenia V. Kastanenka, Miwon Sohn, Sangzin Choi, Song-hyen Choi, HyeYeon Soh, Brian J. Bacskai

**Affiliations:** 1 Department of Neurology, MassGeneral Institute of Neurodegenerative Diseases, Massachusetts General Hospital and Harvard Medical School, 114 Sixteenth St., Charlestown, MA 02129 USA; 2 Dong-A ST, Yongin-Si, Republic of Korea

**Keywords:** Alzheimer’s disease, Multiphoton microscopy, Therapeutic, In vivo, Calcium imaging

## Abstract

**Background:**

Alzheimer’s disease (AD) is a neurodegenerative disorder characterized by deposition of amyloid plaques and disruption of neural circuitry, leading to cognitive decline. Animal models of AD deposit senile plaques and exhibit structural and functional deficits in neurons and neural networks. An effective treatment would prevent or restore these deficits, including calcium dyshomeostasis observed with in-vivo imaging.

**Methods:**

We examined the effects of DA-9803, a multimodal botanical drug, in 5XFAD and APP/PS1 transgenic mice which underwent daily oral treatment with 30 or 100 mg/kg DA-9803 or vehicle alone. Behavioral testing and longitudinal imaging of amyloid deposits and intracellular calcium in neurons with multiphoton microscopy was performed.

**Results:**

Chronic administration of DA-9803 restored behavioral deficits in 5XFAD mice and reduced amyloid-β levels. DA-9803 also prevented progressive amyloid plaque deposition in APP/PS1 mice. Elevated calcium, detected in a subset of neurons before the treatment, was restored and served as a functional indicator of treatment efficacy in addition to the behavioral readout. In contrast, mice treated with vehicle alone continued to progressively accumulate amyloid plaques and calcium overload.

**Conclusions:**

In summary, treatment with DA-9803 prevented structural and functional outcome measures in mouse models of AD. Thus, DA-9803 shows promise as a novel therapeutic approach for Alzheimer’s disease.

**Electronic supplementary material:**

The online version of this article (10.1186/s13195-018-0338-2) contains supplementary material, which is available to authorized users.

## Background

Alzheimer’s disease (AD) is a progressive neurodegenerative disorder and the most prevalent form of dementia [1]. AD is defined by the presence of amyloid plaques and neurofibrillary tangles in the brain [2]. Overwhelming evidence suggests that the aggregation of amyloid-beta (Aβ) initiates a cascade of events that lead to formation of amyloid plaques and neurofibrillary tangles that culminate in loss of neurons [3]. Transgenic mouse models that overexpress human amyloid precursor protein (APP) develop Aβ-related pathologies such as amyloid plaque deposition similar to those in AD patients [4–6]. Additionally, as these mice age, their cortical neurons exhibit alterations in calcium homeostasis, supporting the calcium hypothesis of AD [7–11]. Approximately 20% of neuronal processes exhibit aberrant calcium homeostasis leading to elevated levels of resting calcium, or calcium overload, in APP/PS1 mice aged 8–10 months with substantial amyloid pathology, and this is not related to the presenilin gene [11]. Calcium overload also correlates with disrupted neuronal structure and function [11], so restoring calcium homeostasis could serve as an indirect functional indicator of treatment efficacy. Furthermore, it is well documented that neuroinflammation is central to the progression of the disease, although it is not clear whether it is a cause or a consequence of the disease. An increased number of reactive microglia and astrocytes are concentrated in close proximity to amyloid plaques in humans and mouse models [12–14]. Thus, an effective AD treatment would halt Aβ deposition, restore intraneuronal calcium to control levels, and improve the ability of neuroinflammatory cascades to respond to pathology.

DA-9803 is a multimodal, botanical therapeutic currently in preclinical development by Dong-A ST that shows nootropic promise. It is a well-controlled proprietary extract from *Morus alba L.* and the surface layer of *Poria cocos* that contains multiple major and minor active ingredients. Its pharmacokinetics and mechanism of action are currently under study. The extract is carefully and reproducibly prepared similar to other natural product extracts [15]. Thus, the extract, with no obvious indications of toxicity, is a potential drug candidate for the treatment and prevention of AD.

This work tested the ability of DA-9803 to reverse the structural and functional deficits in two separate AD mouse models. 5XFAD mice show behavioral deficits as they age, which was restored with DA-9803 treatment in parallel with a decrease in Aβ levels measured with ELISA. Multiphoton imaging in a separate cohort of APP/PS1 mice treated with DA-9803 was used to monitor the dynamics of plaque deposition [16] and neuronal calcium levels in the cortex during a 2-month treatment period [16, 17]. Treating young APP/PS1 mice with DA-9803 halted Aβ plaque deposition and decreased the number of neuronal processes exhibiting calcium overload. These results suggest that DA-9803 affects Aβ aggregation as well as functional outcome measures in mouse models, and warrants further investigation of this promising therapeutic.

## Methods

### Animals and surgery

Five-month-old 5XFAD transgenic mice (five males/group) were used for the behavioral analyses. 5XFAD transgenic mice overexpress both mutant human APP(695) with the Swedish (K670N, M671L), Florida (I716V), and London (V717I) mutations as well as human PS1 harboring two FAD mutations, M146L and L286V [6]. The behavioral experiments were approved by Institutional Animal Care and Use Committee of Dong-A ST and conducted according to the IACUC guidelines.

Transgenic APPSwe/PS1dE9 mice were used for longitudinal imaging studies (eight males, five females). APP/PS1 mice overexpress the Swedish mutation in the *APP* gene, as well as the delta E9 mutation in the *PS1* gene [5]. The studies were conducted in compliance with Massachusetts General Hospital Animal Care and Use Committee and NIH guidelines for the use of experimental animals.

At 5 months of age, the APP/PS1 animals underwent intracortical virus injections followed by craniotomies as described previously [18, 19]. Briefly, each animal was anesthetized with 2% isoflurane and placed in a sterotaxic apparatus. Body temperature was maintained with a heating pad throughout the course of anesthesia. The skin over the skull was disinfected with betadine and isopropyl alcohol, and then cut to reveal the skull. Burr holes were drilled through the skull in each hemisphere with the following coordinates: A–P –3 mm, M–L –1 mm, D–V –0.8 mm with respect to the bregma. Using a Hamilton syringe, 1.5 μl of AAV8 Yellow Cameleon 3.6 (YC3.6) (U. Penn Viral Core) was injected 0.8 mm below the dura in both burr holes at a rate of 130 nl/min to target excitatory neurons in the somatosensory cortex. YC3.6 is a genetically encoded ratiometric calcium indicator that reports calcium concentrations quantitatively [11, 20]. A cranial window was then installed by first performing a round craniotomy over the somatosensory cortex. The area over the brain was covered with an 8-mm glass coverslip and fixed with a mixture of crazy glue and dental cement [18, 19, 21]. Each animal remained on a heating pad while recovering from anesthesia. To allow for YC3.6 expression and to limit an inflammatory response associated with the craniotomy interfering with multiphoton imaging, DA-9803 treatment commenced 4 weeks after virus injections and installation of cranial windows. Mice were 6 months old at this point.

### Chronic DA-9803 treatment

Each 5XFAD mouse was assigned randomly to either the vehicle or the DA-9803 treatment condition for the behavioral studies. Then 200 μl of the vehicle or the DA-9803 solution was administered via oral gavage daily. DA-9803 is a botanical drug that can be dissolved in saline solution. The vehicle consisted of phosphate buffered saline (PBS) and hydroxypropyl methyl cellulose (HPMC). The drug treatment was composed of the vehicle solution as well as DA-9803. Animals in the drug condition received doses of either 30 or 100 mg/kg DA-9803. After 4 weeks of daily gavage treatment, the 5XFAD mice were subjected to the Y-maze and the passive avoidance tests. Animals were subsequently euthanized and their brains processed for biochemistry.

For the longitudinal imaging experiments, APP/PS1 animals were assigned randomly to either the vehicle or the DA-9803 treatment condition. Then 300 μl of the vehicle or the DA-9803 solution was administered by oral gavage daily. The vehicle consisted of PBS and HPMC. DA-9803 was added to the vehicle formulation for administration to the treated mice. Animals in the drug condition received a dose of 100 mg/kg DA-9803. Daily gavage treatment commenced after the first multiphoton imaging session. Animals were subsequently imaged 2, 4, and 8 weeks after the first imaging session. Daily gavage treatment was continued until the last imaging session, after which the animals were perfused transcardially with PBS, and their brains extracted and processed for immunohistochemistry. Of the 13 animals that were imaged, seven were treated with the vehicle and six were treated with DA-9803.

### Multiphoton imaging and data acquisition

The day prior to the first imaging session, mice were injected intraperitoneally with 4 mg/kg methoxy-XO4 to label amyloid plaques [11, 22]. Texas Red-labeled dextran was injected via the retro-orbital sinus to provide a fluorescent angiogram. Prior to subsequent imaging sessions, animals were reinjected with methoxy-XO4 to label newly appeared amyloid plaques. Imaging of amyloid plaques and YC3.6-positive neurons was performed using an Olympus Fluoview 1000MPE mounted on an Olympus BX61WI upright microscope. A 25× water immersion objective (NA = 1.05) was used for imaging. A mode-locked titanium:sapphire laser (MaiTai; Spectra-Physics, Fremont, CA, USA) generated two-photon fluorescence with either 800 or 860 nm excitation. Amyloid plaque pathology was imaged using 800 nm excitation at 1× zoom. YC3.6 was imaged using 860 nm excitation at 1×, 2×, and 5× zoom. Laser power was kept below 30 mW to avoid phototoxicity.

After each imaging session, mice were allowed to recover from anesthesia on a heating pad. At the end of the last imaging session, mice were euthanized with CO_2_, perfused with 4% paraformaldehyde in PBS, and fixed with 4% paraformaldehyde and 15% glycerol cryoprotectant overnight. Brains were frozen in Optimal Cutting Temperature compound (OCT), cut into 20-μm coronal sections on a cryostat, and mounted onto slides.

### Image processing and data analysis

Image stacks acquired in vivo were analyzed using ImageJ software (http://rsbweb.nih.gov/ij/). Images were analyzed to determine amyloid plaque numbers, amyloid plaque burden, and resting calcium levels within individual neuronal processes (neurites). The same neurites were followed longitudinally to determine changes in calcium levels in each individual, identified neurite. Neurites that were not present in all four imaging sessions were excluded from analysis. For amyloid plaque analysis, each z-stack was processed into a maximum intensity projection. To determine the amyloid plaque number, amyloid plaques were counted manually in each projected image. To calculate the amyloid burden, each projected image was thresholded, segmented, and the percentage area occupied by amyloid was measured. Any signal from the amyloid lining the blood vessels, cerebral amyloid angiopathy, was excluded from analysis.

YC3.6 images were also analyzed using ImageJ. YC3.6 is a FRET probe, composed of a donor, cyan fluorescent protein (CFP), and an acceptor, yellow fluorescent protein (YFP) [20]. Intraneuronal concentrations were determined from the ratio of YFP to CFP. The higher the calcium concentration, the higher the ratio of YFP to CFP. The background for each channel was calculated by the mode of the intensities of the last slice of each volume and was subtracted from its respective channel. A median filter with a radius of 2 was applied and the fluorescence intensity of YFP was divided by CFP to create a ratio image. Neurites were identified manually and selected using the ‘free hand’ tool on ImageJ in the YFP images to minimize bias based on the calcium concentration. These neuronal regions of interest (ROIs) were then exported to the ratio images and the YFP/CFP ratios calculated. The relative change in YFP/CFP ratio (∆*R*/*R*_*i*_) was calculated by tracking the same neurites throughout all four imaging sessions. YFP/CFP ratios were converted to [Ca^2+^] with standard equations using the in-situ *K*_d_ and Hill coefficient for YC3.6 determined previously [11]. Pseudocolored images were created using Matlab based on the calcium concentration using the empirical *R*_min_ and *R*_max_. The ratio values were used to determine the hue and saturation (color) and the brightness values were used to determine the value (intensity) in the pseudocolored images.

### Immunohistochemistry

Twenty-micrometer transverse coronal sections of mouse brain underwent antigen retrieval in citrate buffer. The coronal sections were then permeabilized with Triton X-100, blocked with normal goat serum (NGS), and incubated with the various primary antibodies: 6E10 (monoclonal 6E10, 1:500; Covance), IBA1 (rabbit anti-Iba1, 1:2000; Wako), glial fibrillary acidic protein (GFAP) (mouse anti-GFAP, 1:200; Thermo Scientific), and MAP2 (mouse anti-MAP2, 1:100; Abcam) for 2 hours at room temperature. The coronal sections were then incubated with the respective secondary antibodies for 1 hour and mounted with ProLong Antifade reagent (Invitrogen).

### Postmortem image analysis

To image amyloid plaque burden post mortem, images of entire cortical hemispheres or hippocampi were acquired using an inverted Zeiss microscope with a 10× objective. Images were analyzed using ImageJ. Methoxy-XO4-labeled plaques were thresholded and the amyloid burden was calculated as a percentage of the cortical (or hippocampal) area.

GFAP, 6E10, MAP2, and IBA1-stained slides were also imaged using an inverted Zeiss microscope at 20× and 40× zoom. The objective was either centered around a single plaque or positioned over an area lacking plaques. Microglia and astrocytes present in the field of view were counted manually. Microglial and astrocytic process length and cell body diameters were analyzed using ImageJ.

### Y-maze test

The Y-maze consisted of three black, opaque, plastic arms (5 cm × 30 cm × 12 cm) 120° from each other. The 5XFAD mice were placed in the center and were allowed to explore all three arms. The number of arm entries and number of triads (a triad constituted subsequent entries into three separate arms) were recorded to calculate the percentage of alternation. An entry was defined as all four appendages entering a Y-maze arm. Alternation behavior was defined as the number of triads divided by the number of arm entries minus 2 and multiplied by 100.

### Passive avoidance test

The passive avoidance chamber was divided into a white (light) and a black (dark) compartment (both 20 cm × 20 cm × 20 cm). The light compartment contained a 50-W electric lamp. The floor of the dark department contained a number of 2-mm stainless steel rods spaced 1 cm apart. Each mouse was placed in the light compartment. The door separating the two compartments was opened 10 seconds later. Once the mouse entered the dark compartment, the door closed and an electrical foot shock (0.1 mA/10 g) was delivered through the grid floor for 3 seconds. Twenty-four hours after the training trial, mice were placed in the light chamber for testing. Latency was defined as the time it took for a mouse to enter the dark chamber after the door separating the two compartments opened.

### Amyloid-beta ELISAs

Total (soluble and insoluble) Aβ40 and Aβ42 levels were detected using the Aβ40 or Aβ42 sandwich ELISA kits. Each 5XFAD cerebral cortex was homogenized in ice-cold buffer and centrifuged at 14,000 × *g* for 30 minutes at 4 °C to isolate Aβ. The resulting supernatants were used for the immunoassay. Standards and tissue samples were incubated in rabbit anti-mouse Aβ40 or Aβ42 polyclonal antibody-precoated 96-well immunoplates overnight at 4 °C (KMB3481 and KMB3441; Invitrogen). The plates were incubated with anti-rabbit IgG HRP for 30 minutes at room temperature. After washing, the plates were incubated with stabilized Chromogen for 30 minutes in the dark. Stop solution was then added to each well. The plates were read at 450 nm using a spectrophotometer (Spectramax microplate reader; Molecular Devices). Concentrations of Aβ40 or Aβ42 were determined in the sample solution using Aβ40 or Aβ42 standard calibration curves. The protein from the samples was normalized using the Bio-Rad assay for total protein determination.

### Statistics

Statistical analyses were conducted in GraphPad 5.0. Data were expressed as mean ± SEM. Datasets were checked for normality (Shapiro–Wilk normality test or Kolmogorov–Smirnov test) and appropriate statistical tests were used (parametric *t* test or ANOVAs for normally distributed data, Mann–Whitney or Kruskal–Wallis test for nonparametric data). *p* < 0.05 was considered significant.

## Results

### One-month daily treatment with 30 mg/kg and 100 mg/kg DA-9803 restores behavioral deficits and decreases Aβ peptide levels in 5XFAD mice

**Fig. 1:**
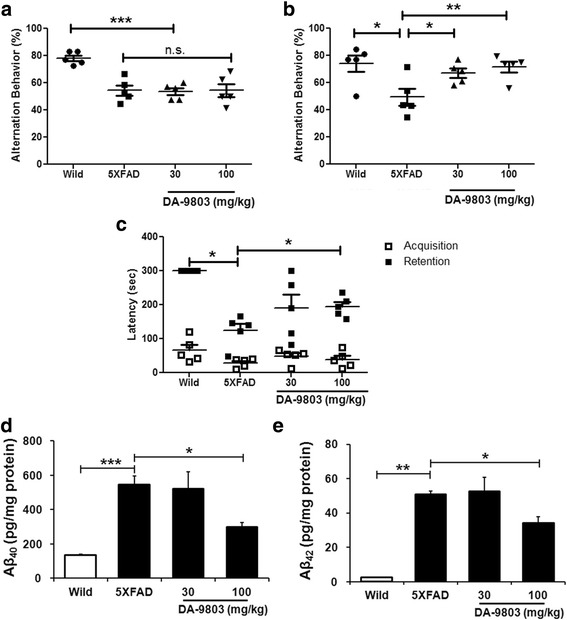
Chronic treatment with DA-9803 restores behavioral deficits and decreases Aβ levels in cortices of 5XFAD mice. **a**, **b** Alternation behavior in a Y-maze before (**a**) and after (**b**) 4-week chronic DA-9803 treatment in 5-month-old 5XFAD mice (*n* = 5 mice/group). **c** Latency of entry into the dark compartment on a passive avoidance test after 4-week DA-9803 treatment (*n* = 5 mice/group). **d**, **e** Total Aβ40 (**d**) and Aβ42 (**e**) levels in cortices of 5XFAD mice after 4-week DA-9803 treatment (*n* = 5 mice/group). Mean ± SEM. *p < 0.05, **p < 0.01, ***p < 0.001. n.s. not significant, Aβ amyloid-beta

5XFAD mice were used to determine the effect of DA-9803 treatment on functional recovery as well as Aβ40 and Aβ42 levels in the cerebral cortex. Mice were treated via daily gavage with the vehicle, 30 mg/kg DA-9803, or 100 mg/kg DA-9803. Animal behavior was monitored using the Y-maze and passive avoidance paradigms. Prior to treatment, 5XFAD mice, regardless of their assigned treatment condition, showed impairment as a function of decreased alternation behavior in the Y-maze, consistent with the literature (Fig. 1a, ANOVA followed by Student–Newman–Keuls method, *n* = 5 mice/group) [6]. After 1 month of treatment, the 30 mg/kg-treated and 100 mg/kg-treated mice showed improvement in alternation behavior between the arms of the Y-maze compared to the vehicle-treated 5XFAD mice (Fig. 1b, ANOVA followed by Student–Newman–Keuls method, *n* = 5 mice/group). Moreover, animals in the 100 mg/kg condition recovered to the levels of wildtype controls (average of 71.7 ± 4.0% and 74.0 ± 6.2% respectively) (Fig. 1b). 5XFAD mice showed impairment in the passive avoidance task as well, spending 124.8 ± 20.3 seconds in the light compartment before returning to the dark compartment when treated with vehicle compared to 300.0 ± 0.0 seconds for the wildtype mice (Fig. 1c, individual *t* tests, *n* = 5 mice/group). The 30 mg/kg-treated and 100 mg/kg-treated mice spent significantly longer periods of time in the light compartment compared to the vehicle-treated mice (189.6 ± 41.1 seconds and 195.0 ± 13.71 seconds respectively compared to 124.8 ± 20.3 seconds), indicative of functional recovery (Fig. 1c). Thus, 1-month 30 mg/kg and 100 mg/kg DA-9803 treatment improves the behavioral deficit in the Y-maze and the passive avoidance task in 5XFAD mice, suggesting that DA-9803 has beneficial effects in these animals.

To investigate the effect of DA-9803 treatment on Aβ, the Aβ40 and Aβ42 levels were measured using ELISAs in 5XFAD mice. Aβ40 and Aβ42 analyses showed similar trends. Aβ40 and Aβ42 levels were the lowest in wildtype mice (134.6 ± 60 pg/mg and 2.44 ± 0.24 pg/mg respectively) (Fig. 1d, e). Vehicle-treated and 30 mg/kg-treated mice had comparable levels of Aβ40 and Aβ42 after treatment (vehicle-treated mice: 546.4 ± 48.8 pg/mg of Aβ40 and 50.7 ± 2.1 pg/mg of Aβ42; 30 mg/kg-treated mice: 521.4 ± 99.9 pg/mg of Aβ40 and 52.6 ± 8.3 pg/mg of Aβ42) (Fig. 1d, e). The 100 mg/kg treatment significantly lowered the levels of Aβ40 and Aβ42 compared to the vehicle (297.3 ± 26.7 pg/mg of Aβ40 and 34.1 ± 3.9 pg/mg of Aβ42, ANOVA followed by Newman–Keuls post-hoc test, *n* = 5 cortices/group) (Fig. 1d, e). These data suggest that DA-9803 is reducing production or increasing clearance of Aβ40 and Aβ42 via a dose-dependent mechanism.

### Two-month daily treatment with DA-9803 halts plaque deposition in-vivo in APP/PS1 mice

**Fig. 2:**
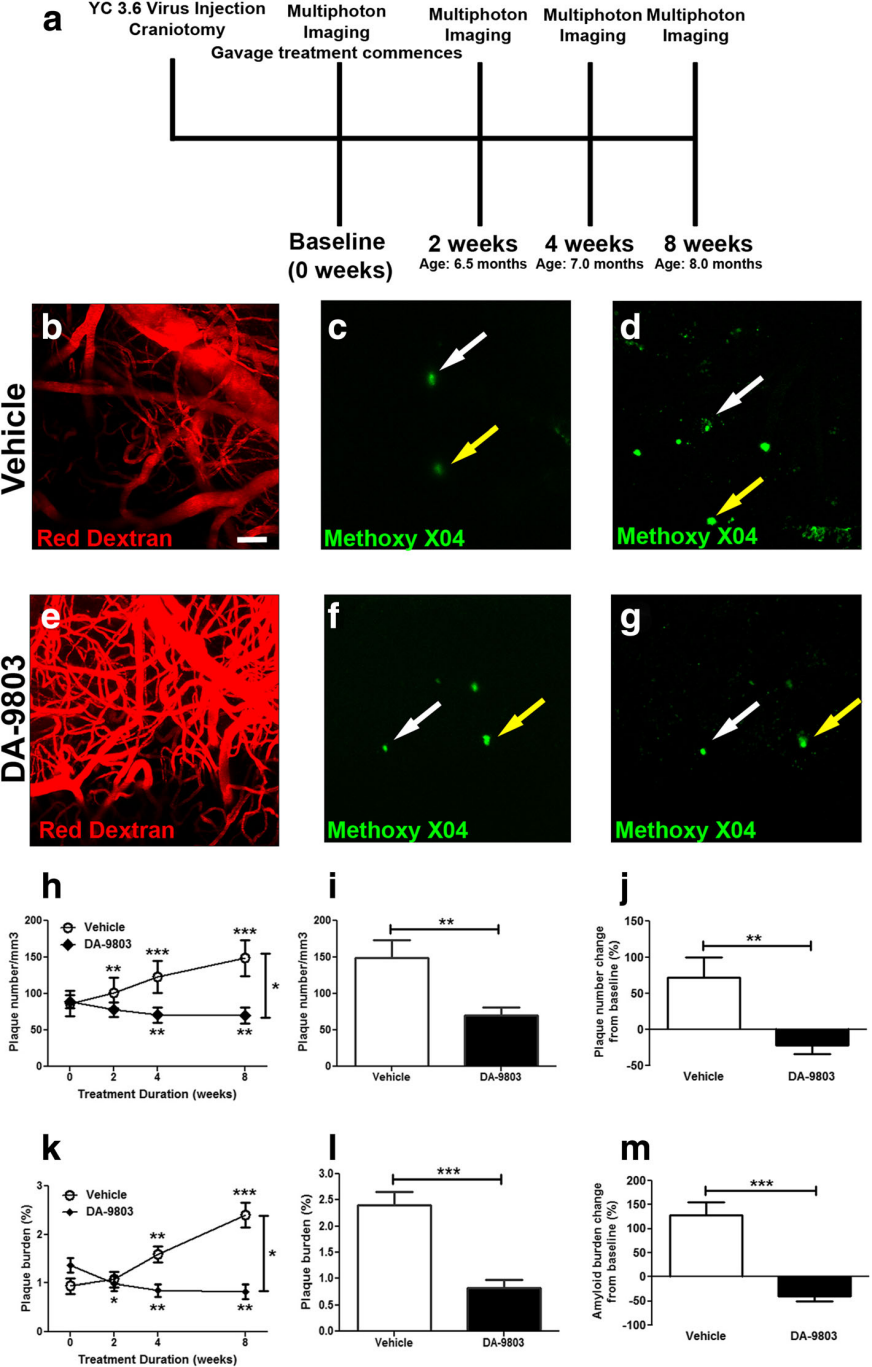
Chronic treatment with DA-9803 slows plaque deposition and results in mild clearance of amyloid plaques in APP/PS1 mice. **a** Protocol for chronic gavage treatment of DA-9803 (*n* = 6) or control compound (*n* = 6) in 6-month-old APP/PS1 mice. Methoxy-XO4 injected intraperitoneally (IP) before each imaging session. **b**–**d** Two-photon images of an angiogram (**b**) and of methoxy-positive amyloid plaques taken at baseline (**c**) and after 8 weeks of treatment (**d**) of a mouse treated with control compound. **e**–**g** Two-photon images of an angiogram (**e**) and of methoxy-positive amyloid plaques taken at baseline (**f**) and after 8 weeks of treatment (**g**) of a mouse treated with DA-9803. **h**, **k** Amyloid plaque number (**h**) and amyloid plaque burden (**k**) during the course of 8-week treatment across conditions. Amyloid plaque burden and plaque number values compared to baseline values via nonparametric Mann–Whitney test. **i**, **l** Amyloid plaque number (**i**) and amyloid plaque burden (**l**) after 8 weeks of treatment across conditions. **j**, **m** Change in amyloid plaque number (**j**) and burden (**m**) at the end of treatment compared to baseline. Scale bar, 100 μm. Mean ± SEM. *p < 0.05, **p < 0.01, ***p < 0.001

APP/PS1 mice were used to monitor the longitudinal effect of DA-9803 treatment on plaque deposition. Mice were treated via daily gavage with either the vehicle or 100 mg/kg DA-9803 for 2 months (Fig. 2a). Individual plaques were imaged over time with multiphoton microscopy (Fig. 2b-g). Angiograms were used as fiducial markers to find the plaques in the same locations over time (Fig. 2b, e). Mice in the vehicle and the drug conditions started with a similar number of plaques in the somatosensory cortex: vehicle-treated mice exhibited 86 ± 17 plaques/mm^3^ while DA-9803-treated mice had 89 ± 9 plaques/mm^3^ (mean ± SEM) at baseline (Fig. 2h). Throughout the 2-month treatment period, animals treated with the vehicle exhibited an increase in the number of amyloid plaques accumulating to an average of 140 ± 45 plaques/mm^3^ by the end of treatment, statistically different from baseline (mean ± SEM, Mann–Whitney test *p* < 0.001, *n* = 33 z-stacks in six mice treated with vehicle) (Fig. 2h). Conversely, animals treated with DA-9803 exhibited a decrease in amyloid plaque number and thus showed both prevention of plaque deposition and plaque clearance (average of 67 ± 20 plaques/mm^3^ at the end of DA-9803 treatment, significantly different from baseline, Mann–Whitney test *p* < 0.01, *n* = 30 z-stacks in six mice treated with DA-9803) (Fig. 2h). Thus, the rates of amyloid plaque deposition were different between the two conditions (simple linear regression *p* < 0.05) (Fig. 2h). By the end of treatment, the number of amyloid plaques was significantly lower in DA-9803-treated compared to vehicle-treated animals (Fig. 2i). Likewise, the amyloid burden, which was similar at the beginning of treatment, increased in the vehicle condition and decreased in the drug condition (Fig. 2k). Hence, amyloid plaque burden was significantly lower after the DA-9803 treatment compared to the vehicle (Mann–Whitney test *p* < 0.001, *n* = 30 z-stacks in six mice treated with DA-9803, *n* = 33 z-stacks in six mice treated with vehicle; Fig. 2l). Therefore, DA-9803-treated mice exhibited a 21.6 ± 11.94% reduction in amyloid plaque number compared to baseline, while vehicle-treated mice presented a 72.13 ± 27.85% increase (Fig. 2j). Similarly, amyloid plaque burden decreased by the end of the DA-9803 treatment compared to baseline, while increasing in the vehicle condition (Fig. 2m). Thus, daily oral treatment with DA-9803 halted plaque deposition and led to a reduction in amyloid plaque number and amyloid plaque burden in APP/PS1 mice over the 2-month period.

**Fig. 3:**
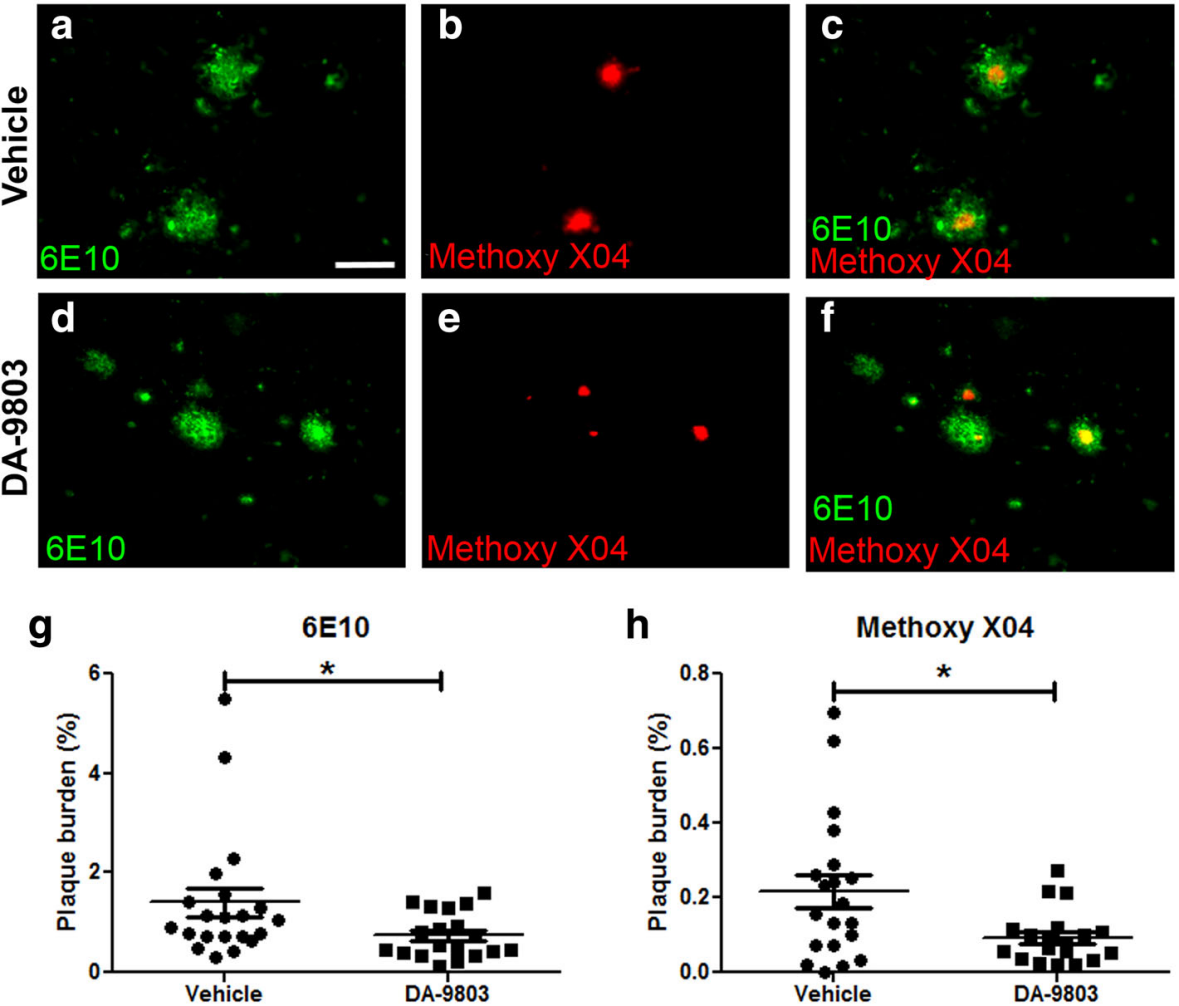
DA-9803-treated mice had less postmortem cortical Aβ plaque load than vehicle-treated APP/PS1 mice. **a**–**c** Confocal microscope images of 6E10-positive (**a**), methoxy-XO4-labeled (**b**), and colabeled (**c**) plaques in the vehicle condition. **d**–**f** Confocal microscope images of 6E10-stained (**d**), methoxy-XO4-labeled (**e**) and colabeled (**f**) plaques in the vehicle condition. **g** Percentage of cortex occupied by 6E10-positive Aβ across conditions. **h** Percentage of cortex occupied by methoxy-XO4-positive plaques across conditions. Scale bar, 100 μm. Mean ± SEM. *p < 0.05

To confirm the in-vivo findings, immunohistochemistry with the anti-Aβ antibody 6E10 was performed on the 20-μm thick coronal sections of brains isolated from animals that underwent amyloid imaging in vivo. Amyloid plaque burden was visualized ex vivo with the methoxy-XO4 signal that remained from in-vivo imaging as well as with 6E10. Methoxy-XO4 labeling colocalized with 6E10 immunostaining in vehicle-treated animals (Fig. 3a–c) and those treated with DA-9803 (Fig. 3d-f). Methoxy-XO4 labeled dense core plaques, while 6E10 immunolabeled the Aβ-including and surrounding dense core plaques (Fig. 3c, f), similar to earlier reports [22, 23]. We analyzed 6E10-labeled and methoxy-XO4-labeled amyloid burden in the cortices of mice treated with DA-9803 and the vehicle (*n* = 19 cortical sections in six mice treated with DA-9803, *n* = 20 cortical sections in six mice treated with vehicle). The amyloid burden detected with 6E10 in DA-9803-treated mice was significantly lower than the amyloid burden in vehicle-treated mice (0.75 ± 0.10% plaque load vs 1.4 ± 0.28% plaque load, Mann–Whitney test *p* < 0.05) (Fig. 3g). Likewise, the amyloid burden detected with methoxy-XO4 in DA-9803-treated mice was significantly lower than the amyloid burden in vehicle-treated mice in the cortex (0.09 ± 0.02% plaque load vs 0.22 ± 0.04% plaque load, Mann–Whitney test *p* < 0.05) (Fig. 3h) and the hippocampus (0.03 ± 0.01% plaque load vs 0.14 ± 0.03% plaque load) (Additional file 2: Figure S2). These data confirm the results from the in-vivo imaging experiments.

To test whether DA-9803 or vehicle interfered chemically with methoxy-XO4 binding to plaques, fixed postmortem brain sections containing plaques were preincubated with either 10 mg/ml DA-9803, vehicle, or PBS, each followed by incubation with 4 mg/kg methoxy-XO4. The fluorescence intensity of methoxy-XO4 was comparable in all three conditions, indicating that DA-9803 did not interfere with methoxy-XO4 binding to amyloid plaques (Additional file 1: Figure S1). These data suggest that DA-9803 had a biological effect on amyloid plaque clearance rather than a direct chemical interference preventing visualization of amyloid plaques during imaging.

### Chronic treatment decreases the number of neurites with calcium overload in APP/PS1 mice

**Fig. 4:**
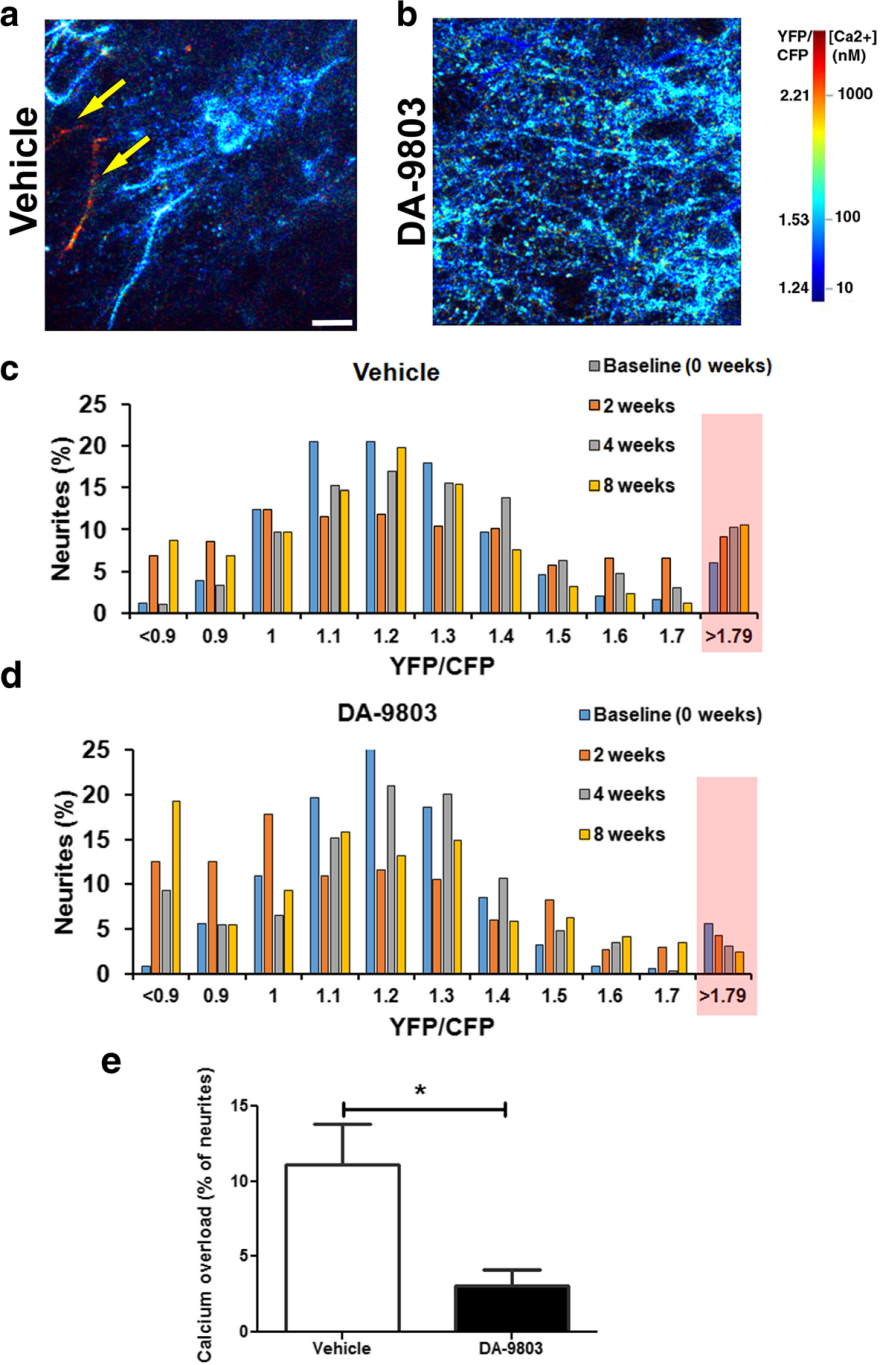
Calcium overload in DA-9803-treated mice is significantly decreased after 8 weeks of treatment. **a**, **b** Two-photon images of cortical neurites, pseudocolored according to neuronal [Ca^2+^] concentration and intensity, in vehicle-treated (**a**) and DA-9803-treated (**b**) mice. Red neurites exhibit elevated levels of calcium, blue neurites exhibit normal calcium levels. Yellow arrows denote overloaded neurites. **c**, **d** Histograms of neurite YC3.6 ratios for APP/PS1 mice in the vehicle (**c**) and DA-9803 (**d**) conditions. *n* = 361 neurites in six mice in vehicle condition. *n* = 290 neurites in five mice in DA-9803 condition. **e** Percentage of neurites exhibiting calcium overload across conditions after 8 weeks of treatment. Scale bar, 10 μm. Mean ± SEM. *p < 0.05. YFP yellow fluorescent protein, CFP cyan fluorescent protein

Calcium homeostasis is necessary for proper neuronal function. Aberrant levels of intraneuronal calcium have been linked to altered APP processing, synaptic dysfunction, and AD symptoms in humans [24]. A higher proportion of neurons exhibit elevated levels of calcium (calcium overload) in aged APP/PS1 mice compared to wildtype littermates [11]. We analyzed neuronal calcium in vivo using the calcium sensor Yellow Cameleon 3.6 (YC3.6) to determine whether chronic DA-9803 treatment would restore the calcium levels to normal in neuronal processes, or neurites, exhibiting calcium overload. Elevated calcium levels or calcium overload was defined as calcium concentrations two standard deviations greater than the average calcium concentration within the neurons in wildtype mice. Thus, a neuron with YFP/CFP ratio greater than 1.79, and a respective calcium concentration greater than 235 nM, was defined to exhibit calcium overload (Fig. 4a, yellow arrows pointing to red neurites). At baseline, prior to treatment, 6% of neurites in the drug group and 6% of neurites in the vehicle group exhibited calcium overload (Fig. 4c, d, see red boxes).

We collected additional resting calcium data during the subsequent imaging sessions, at 2, 4, and 8 weeks after treatment onset. Fluorescent angiograms were used to provide fiduciary markers to ensure the same individual neurites were identified across imaging sessions. After 2 months of treatment, the percentage of neurites with calcium overload decreased to 2% of neurites in mice treated with DA-9803 (Fig. 4b, d, e). Conversely, the percentage of overloaded neurites increased to 11% in mice treated with vehicle alone (Fig. 4a, c, e). Since the same neurites were tracked throughout the 2 months, the decrease in the number of overloaded neurites was a result of a genuine calcium restoration and not a result of neurite loss. Therefore, these data suggest that chronic treatment with DA-9803 selectively rescued overloaded neurites by restoring their baseline calcium and prevented additional neurites from exhibiting calcium overload.

### Chronic treatment with DA-9803 induces morphological transformation in astrocytes and microglia in APP/PS1 mice

**Fig. 5:**
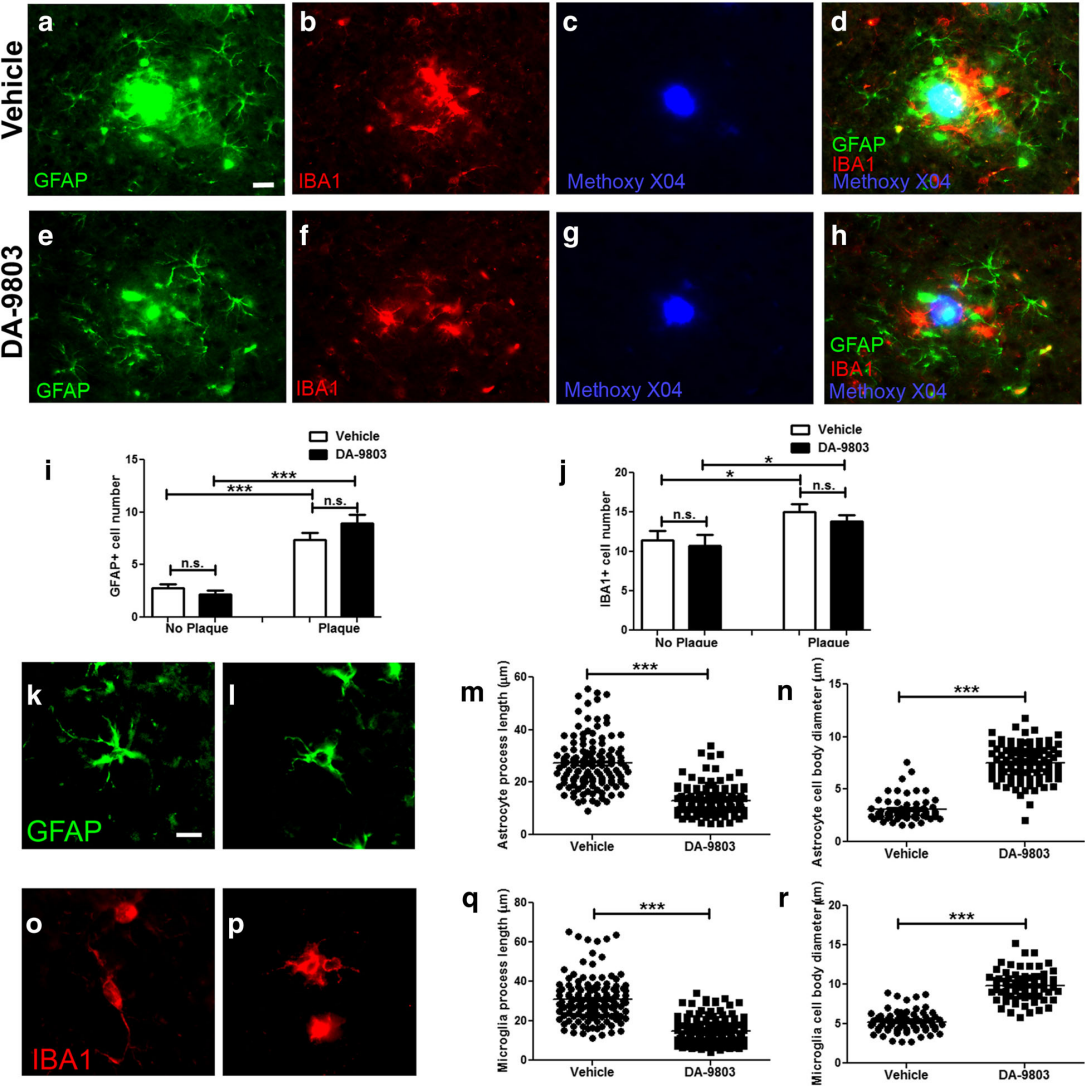
IBA1 and GFAP immunoreactivity after treatment with DA-9803 and vehicle. **a**–**d** Fluorescent images of GFAP-positive astrocytes (**a**), IBA1-positive microglia (**b**), methoxy-XO4-labeled plaques (**c**), and colocalization of GFAP, IBA1, and methoxy-XO4 (**d**) in APP/PS1 mice treated with vehicle compound. **e**–**h** Fluorescent microscope images of GFAP-positive astrocytes (**e**), IBA1-positive microglia (**f**), methoxy-XO4-labeled plaques (**g**), and colocalization of GFAP, IBA1, and methoxy-XO4 (**h**) in APP/PS1 mice treated daily with 100 mg/kg DA-9803. Scale bar, 20 μm. **i** Average number of astrocytes in close proximity (plaque) and away from plaques (no plaque) in a field of view across conditions. **j** Average number of microglia in close proximity (plaque) and away from plaques (no plaque) in a field of view across conditions. **k**, **l** High-magnification fluorescent images of GFAP-positive astrocytes treated with vehicle (**k**) and DA-9803 (**l**). **m** Astrocyte process length across conditions. **n** Astrocyte cell body diameter across conditions. **o**, **p** High magnification fluorescent images of IBA1-positive microglia treated with vehicle (**o**) and DA-9803 (**p**). **q** Microglial process length across conditions. **r** Microglial cell body diameter across conditions. Scale bar, 10 μm. Mean ± SEM. *p < 0.05, ***p < 0.001. n.s. not significant, GFAP glial fibrillary acidic protein

Activation of astrocytes and microglia has been detected in brains from AD patients and APP/PS1 mice [12, 14, 25]. To determine the effect of DA-9803 on astrogliosis, 20-μm postmortem brain sections of the vehicle-treated and DA-9803-treated mice were immunostained with anti-glial fibrillary acidic protein (GFAP) antibody (Fig. 5a, d, e, h) [26]. The number of GFAP-positive astrocytes in the cortex of these animals did not differ between vehicle-treated and DA-9803-treated mice (Fig. 5i). However, reactive astrocytes in both conditions were more likely to cluster around plaques, as described previously (Fig. 5i) [27]. The brain sections were also immunostained with an anti-Iba1 antibody to examine microglial reactivity (Fig. 5b, d, f, h) [28]. We found no difference in Iba1-positive microglia number in DA-9803-treated and vehicle-treated animals (Fig. 5j). The number of microglia was increased in close proximity to plaques (Fig. 5j). MAP2 immunostaining revealed no obvious changes in neuronal morphology or any indication of toxicity (Additional file 2: Figure S2).

To determine the extent of astrocytic and microglial activation, we performed quantitative analysis of glial morphology. When treated with vehicle, astrocytes exhibited lengthy processes and small cell bodies (Fig. 5k-n). In contrast, DA-9803 treatment resulted in shortening of astrocytic processes and enlargement of cell bodies, consistent with activation of astrogliosis [29] (Fig. 5l-n). Similarly, DA-9803-treated microglia exhibited shortening of their processes and an increase in the cell body diameter (Fig. 5o-r), potentially suggesting a transition to a phagocytic state [30, 31].

These results suggest that DA-9803 induces activation of astrocytes and microglia, and possibly transitions glia to a state prone to Aβ phagocytosis and its clearance.

## Discussion

We assessed the effects of a multimodal botanical extract, DA-9803, on behavioral deficits in 5XFAD mice as well as amyloid pathology, neuronal calcium homeostasis, and neuroinflammation in APP/PS1 mice. DA-9803 is a therapeutic candidate currently in preclinical development for the treatment or prevention of AD, with no obvious indications of toxicity. The study followed two protocols. First, 5XFAD animals underwent a 1-month gavage treatment with DA-9803 or vehicle control daily. Behavior was assessed using passive avoidance and Y-maze paradigms. ELISAs were used to determine Aβ40 and Aβ42 levels in the cortex. In the second protocol, APP/PS1 animals were treated for 2 months with daily oral treatment of DA-9803 or vehicle control. Multiphoton microscopy was used to monitor plaques and intraneuronal calcium levels longitudinally in the animals. Postmortem plaque load and neuroinflammation were also assessed. Chronic DA-9803 treatment reversed the behavioral impairments observed in 5XFAD mice. It also decreased Aβ40 and Aβ42 levels as well as the plaque load in the cortex. The percentage of neurites that exhibited high levels of intracellular calcium was lower in drug-treated compared to the control-treated animals. Furthermore, DA-9803 treatment led to morphological changes in astrocytes and microglia suggesting a transition to a phagocytic state.

Five-month-old 5XFAD mice were tested using the Y-maze and passive avoidance techniques after 1-month 30 mg/kg and 100 mg/kg DA-9803 treatment. The animals that were treated with the vehicle alone were significantly impaired in both paradigms and had elevated levels of Aβ40 and Aβ42 compared to the wildtype mice. Both the 30 mg/kg-treated and the 100 mg/kg-treated animals performed better than their vehicle-treated counterparts on the behavioral tests. Moreover, there was no statistical difference between the 30 mg/kg-treated and 100 mg/kg-treated animals, implying that the 30 mg/kg dose was sufficient for behavioral improvement. Interestingly, 100 mg/kg treatment reduced cortical Aβ40 and Aβ42 levels while 30 mg/kg treatment failed to do so. These results indicate that besides targeting Aβ40 and Aβ42, DA-9803 could affect additional pathways that led to improvement in the behavioral tests, supporting the multimodal nature of DA-9803.

Individual plaques were tracked longitudinally during four separate imaging sessions throughout the 2-month drug treatment in the APP/PS1 animals. Mice treated with the vehicle alone exhibited an increase in both amyloid plaque number and burden over time, characteristic of this amyloidosis model [32]. The DA-9803-treated mice developed a limited number of new plaques in the course of the 2-month treatment compared to the vehicle-treated animals. This resulted in the DA-9803-treated animals exhibiting a lower plaque number and burden compared to the vehicle-treated mice at the end of the treatment. This result was confirmed in postmortem coronal sections of brain on a more global scale with measures of both total cortical and hippocampal amyloid plaque burden. These findings complement the immunoassay results in the 5XFAD mice exhibiting lower levels of Aβ40 and Aβ42 after 100 mg/kg DA-9803 treatment. Taken together, we demonstrated that DA-9803 treatment rescued behavioral deficits, prevented progression of amyloid plaque deposition, and led to clearance of Aβ in transgenic mouse models of AD.

The mechanisms by which DA-9803 prevented plaque deposition and/or promoted plaque clearance was unknown. DA-9803 could have inhibited Aβ aggregation or synthesis. Alternatively, DA-9803 could have accelerated degradation or clearance of Aβ. Regardless of the exact mechanisms, targeting Aβ could prevent/slow progression of AD, since it is likely that Aβ initiates the cascade of AD pathology [33–35]. Aβ clearance could explain the decreased plaque load in the DA-9803-treated animals. The current work, however, began with young mice exhibiting sparse plaque load, and instances of plaque clearance were rare. Future studies analyzing the effects of DA-9803 in older transgenic animals with considerable plaque load will be instrumental in testing the treatment efficacy of DA-9803 as opposed to a prevention measure for AD that was tested in the present study. Also, the mechanisms of action of DA-9803 will be investigated in future studies.

In addition, DA-9803’s effect on calcium homeostasis was assessed in vivo. Aβ has been shown to disrupt calcium homeostasis that resulted in neuronal calcium elevations [11]. Hence, restoration of intraneuronal calcium serves as an indirect functional readout of treatment efficacy. Longitudinal imaging with multiphoton microscopy allowed us to track individually identified neurites as these neurites began or ceased exhibiting calcium overload. The percentage of neurites with calcium overload in the DA-9803-treated animals decreased throughout all four imaging sessions while the vehicle-treated animals continued to accumulate neurites exhibiting calcium overload during this period. The mechanism by which DA-9803 decreased calcium overload is unclear. However, it is plausible to speculate that DA-9803 neutralized the toxic Aβ species, resulting in decreased Aβ40 and Aβ42 levels and a reduction in amyloid plaque load as described earlier, ultimately leading to decreased calcium overload. DA-9803 treatment could be affecting processes downstream of calcium homeostasis, such as restoration of calcium compartmentalization, calcineurin activity, and/or the transcriptional factor nuclear factor of activated T cells (NFAT) localization, all of which have been reported previously to be disrupted by elevated calcium [36].

Neuroinflammation is believed to be central to the progression of AD. Modulating the reactivity of astrocytes and microglia could lead to increased phagocytosis and clearance of Aβ as well as other neurotoxic species in the CNS. This activity could lead to the prevention of amyloid deposition, increased clearance, and possible downstream effects on neural function that would include calcium overload, neuronal degeneration, and deficits in behavioral tasks. We performed immunohistochemical analyses of reactive glia in the brains of mice used in the longitudinal imaging experiments. Activation of astrocytes and microglia especially in close proximity to amyloid plaques have been reported in animal models of AD as well as brain tissue of AD patients [14, 25, 37]. Activation of astrocytes and microglia, specifically around amyloid plaques, were detected in the current study. However, there was no statistical difference in the number of either reactive astrocytes or microglia in the drug-treated animals compared to vehicle-treated mice. However, further analysis revealed morphological alterations of astrocytes and microglia after DA-9803 treatment. Shortening of the processes and increases in cell body diameter could indicate a glial transition to a phagocytic state [29–31], possibly rendering them more prone to clearing Aβ by internalization of the toxic peptide [38]. This suggests that chronic treatment of DA-9807 increases a neuroinflammatory profile that may contribute to Aβ clearance through phagocytosis by glial cells.

## Conclusions

In summary, treatment with the botanical therapeutic DA-9803 restored behavioral deficits, prevented amyloid deposition, and decreased Aβ40 and Aβ42 levels in the cortex. The treatment also restored calcium homeostasis and possibly facilitated neuroinflammation which could lead to enhanced clearance of Aβ. DA-9803 is a complex extract containing multiple bioactive ingredients, and as such is likely to have multiple mechanisms of action. The current work demonstrates that DA-9803 shows promise in preventing or alleviating AD pathology and pathophysiology. Future studies will be directed toward characterization of the bioactive components, pharmacokinetics, potency, mechanism(s) of action, and toxicity. Considering the robust effects in the animal models, we believe that further examination of DA-9803 is warranted as a candidate therapeutic approach in AD.

## Additional files

Additional file 1: Figure S1. Showing DA-9803 does not chemically interfere with the binding of methoxy-XO4 to amyloid plaques. **A–C** Fluorescent images of methoxy-XO4 amyloid plaques after preincubation with (**A**) DA-9803 (27 sections from one APP/PS1 mouse), (**B**) PBS (30 sections from one mouse), and (**C**) vehicle compound (29 sections from one mouse). **D** Fluorescence intensity of individual amyloid plaques across conditions. Scale bar, 100 μm. Mean ± SEM. (PDF 748 kb)

Additional file 2: Figure S2. Showing methoxy-XO4 stained amyloid plaque burden and MAP2 immunoreactivity in postmortem hippocampus after treatment with vehicle or DA-9803. **A–C** Fluorescent images of MAP2-positive neurons **(A)**, methoxy-XO4-labeled plaques (**B**), and colocalization of MAP2 and methoxy-XO4 (**C**) in APP/PS1 mice treated daily with the vehicle compound (*n* = 14 sections from seven mice). **D–F** Fluorescent images of MAP2-positive neurons (**D**), methoxy-XO4-labeled plaques (**E**), and colocalization of MAP2 and methoxy-XO4 **(F)** in APP/PS1 mice treated daily with 100 mg/kg DA-9803 (*n* = 17 sections from five mice). Scale bar, 100 μm. **G** Percentage of cortex occupied by methoxy-XO4-positive plaques across conditions. Mean ± SEM. *p < 0.05. (PDF 2111 kb)
