# Supplementary figures and images for: Novel botanical drug DA-9803 prevents deficits in Alzheimer’s mouse models
Source: Alzheimers Res Ther. 2018 Jan 29;10:11. doi: 10.1186/s13195-018-0338-2 (PMC5789736; doi:10.1186/s13195-018-0338-2)

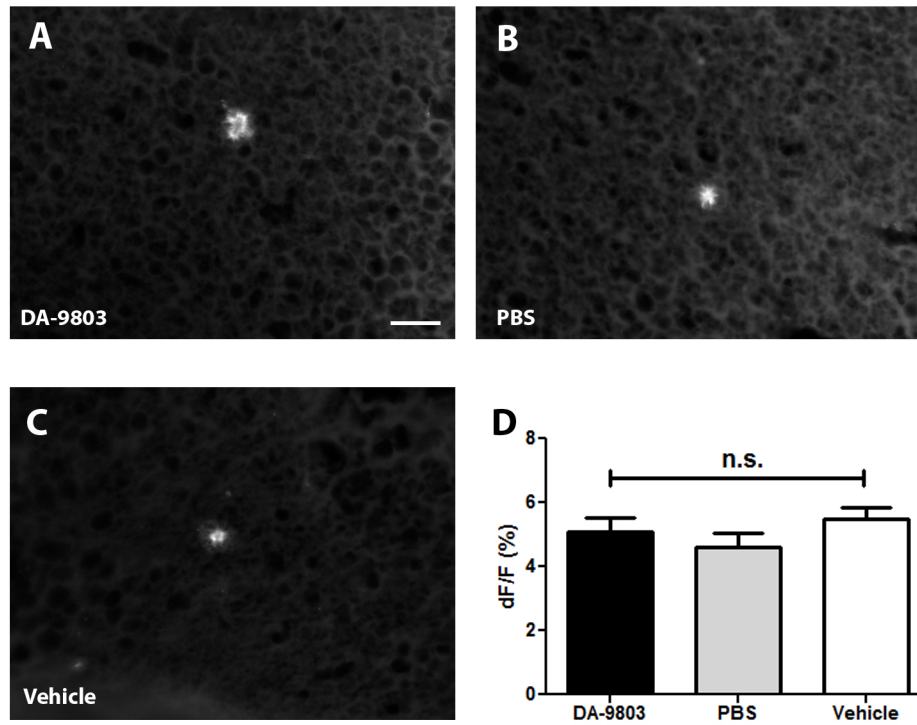

Supplemental Figure 1

Supplement: Supplementary file 1 — Showing DA-9803 does not chemically interfere with the binding of methoxy-XO4 to amyloid plaques. A–C Fluorescent images of methoxy-XO4 amyloid plaques after preincubation with (A) DA-9803 (27 sections from one APP/PS1 mouse), (B) PBS (30 sections from one mouse), and (C) vehicle compound (29 sections from one mouse). D Fluorescence intensity of individual amyloid plaques across conditions. Scale bar, 100 μm. Mean ± SEM. (PDF 748 kb) [file 13195_2018_338_MOESM1_ESM.pdf]
