# Supplementary material for: Novel botanical drug DA-9803 prevents deficits in Alzheimer’s mouse models
Source: Alzheimers Res Ther. 2018 Jan 29;10:11. doi: 10.1186/s13195-018-0338-2 (PMC5789736; doi:10.1186/s13195-018-0338-2)
Supplement: Supplementary file 2 — Showing methoxy-XO4 stained amyloid plaque burden and MAP2 immunoreactivity in postmortem hippocampus after treatment with vehicle or DA-9803. A–C Fluorescent images of MAP2-positive neurons (A), methoxy-XO4-labeled plaques (B), and colocalization of MAP2 and methoxy-XO4 (C) in APP/PS1 mice treated daily with the vehicle compound (n = 14 sections from seven mice). D–F Fluorescent images of MAP2-positive neurons (D), methoxy-XO4-labeled plaques (E), and colocalization of MAP2 and methoxy-XO4 (F) in APP/PS1 mice treated daily with 100 mg/kg DA-9803 (n = 17 sections from five mice). Scale bar, 100 μm. G Percentage of cortex occupied by methoxy-XO4-positive plaques across conditions. Mean ± SEM. *p < 0.05. (PDF 2111 kb) [file 13195_2018_338_MOESM2_ESM.pdf]

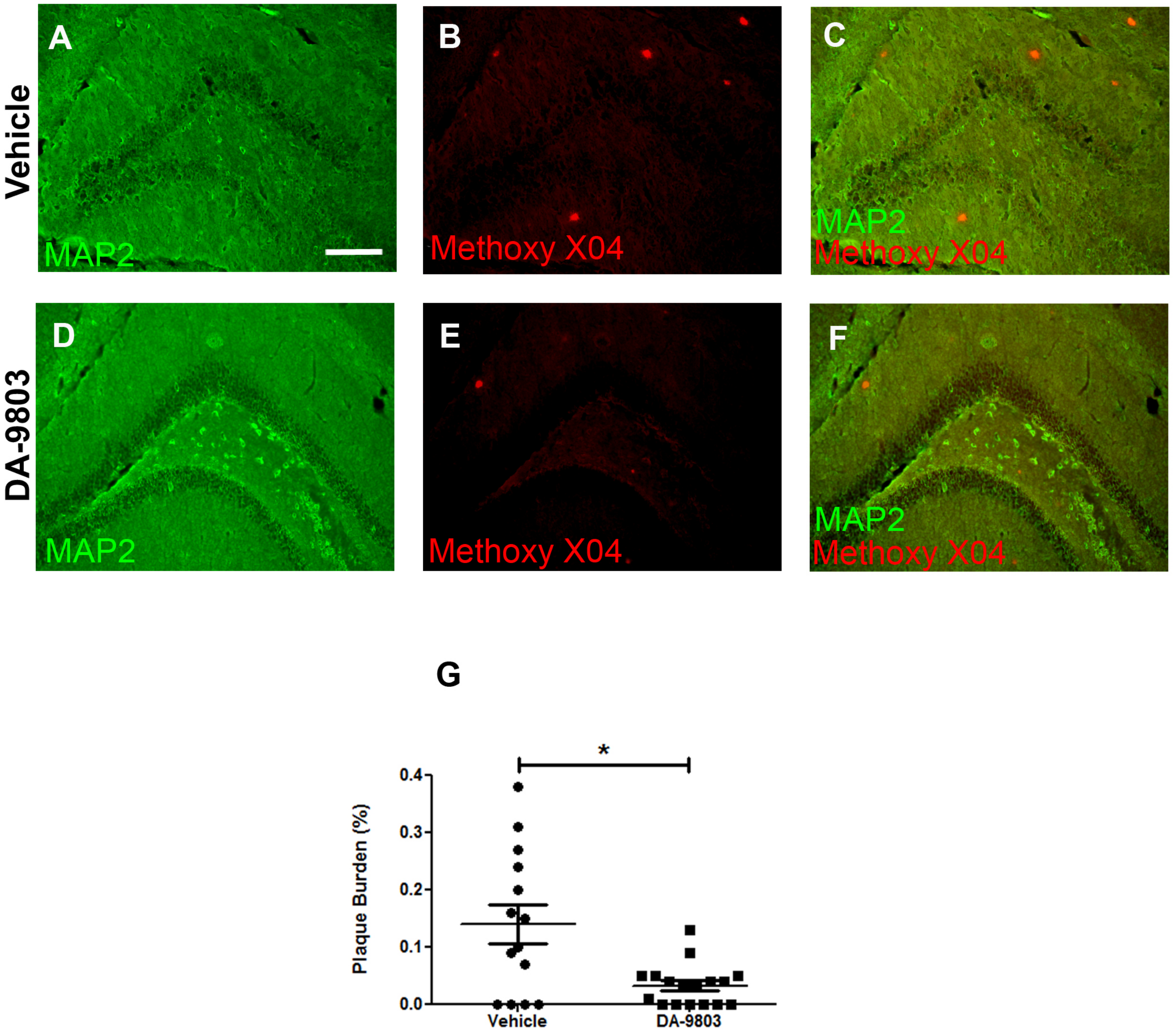

Supplemental Figure 2
